# *Bordetella pertussis* pertactin knock-out strains reveal immunomodulatory properties of this virulence factor

**DOI:** 10.1038/s41426-018-0039-8

**Published:** 2018-03-21

**Authors:** Elise Sofie Hovingh, Rob Mariman, Luis Solans, Daniëlle Hijdra, Hendrik-Jan Hamstra, Ilse Jongerius, Marjolein van Gent, Frits Mooi, Camille Locht, Elena Pinelli

**Affiliations:** 1 Centre for Infectious Disease Control, National Institute for Public Health and the Environment (RIVM), 3721 MA Bilthoven, The Netherlands; 2 Department of Medical Microbiology, University Medical Centre Utrecht, 3584 CX Utrecht, The Netherlands; 3 Centre for Infection and Immunity, Institute Pasteur de Lille, 59000 Lille, France

## Abstract

Whooping cough, caused by *Bordetella pertussis*, has resurged and presents a global health burden worldwide. *B. pertussis* strains unable to produce the acellular pertussis vaccine component pertactin (Prn), have been emerging and in some countries represent up to 95% of recent clinical isolates. Knowledge on the effect that Prn deficiency has on infection and immunity to *B. pertussis* is crucial for the development of new strategies to control this disease. Here, we characterized the effect of Prn production by *B. pertussis* on human and murine dendritic cell (DC) maturation as well as in a murine model for pertussis infection. We incubated human monocyte-derived DCs (moDCs) with multiple isogenic Prn knockout (Prn-KO) and corresponding parental *B. pertussis* strains constructed either in laboratory reference strains with a Tohama I background or in a recently circulating clinical isolate. Results indicate that, compared to the parental strains, Prn-KO strains induced an increased production of pro-inflammatory cytokines by moDCs. This pro-inflammatory phenotype was also observed upon stimulation of murine bone marrow-derived DCs. Moreover, RNA sequencing analysis of lungs from mice infected with *B. pertussis* Prn-KO revealed increased expression of genes involved in cell death. These in vitro and in vivo findings indicate that *B. pertussis* strains which do not produce Prn induce a stronger pro-inflammatory response and increased cell death upon infection, suggesting immunomodulatory properties for Prn.

## Introduction

*Bordetella pertussis* is a Gram-negative respiratory pathogen that causes pertussis, also known as whooping cough, a vaccine-preventable disease. Despite high vaccination coverage, pertussis has been re-emerging in many countries in recent decades and remains a public health problem^1^. This highly contagious respiratory disease can be life threatening in unvaccinated infants and causes significant morbidity across all age groups^2^. Towards controlling this disease, two different types of pertussis vaccines are licensed, namely whole cell vaccines (WCV) consisting of inactivated bacteria and acellular vaccines (ACV) consisting of one to five bacterial antigens^3,4^. In the national immunization programs of most Western countries, including the Netherlands, ACVs have replaced WCVs to minimize adverse effects^5^. These ACVs contain the highly immunogenic virulence factor pertactin (Prn).

In recent years, the emergence of *B. pertussis* strains lacking Prn has been observed, especially in countries using the ACVs, with the percentage of Prn-deficient strains rising over time^6,7^. Prn is a 93 kDa autotransporter protein of *B. pertussis* commonly referred to as a minor adhesion, this however, remains controversial^8–10^. As for other autotransporter proteins, Prn is synthesized as a precursor and further processed to a 30 kDa channel and 69 kDa passenger domain^11^. The passenger domain is transported through the channel across the outer membrane, where it can be displayed at the bacterial surface or cleaved off. The role of Prn during infection is not fully understood, nor is the effect that Prn deficiency in this bacterium has on the host immune response. In a previous study we compared the effect of Prn-producing and Prn-deficient clinical isolates on human monocyte-derived dendritic cell (moDC) maturation and found no differences^12^. Since we cannot rule out that other mutations in the bacterial genome compensate for the loss of Prn, comparing the effect of these naturally circulating *B. pertussis* strains does not necessarily provide information on the effect of Prn only.

Here, we used both human monocyte-derived dendritic cells (moDCs) and murine bone marrow-derived DCs (BMDC) incubated with various isogenic parental or Prn knockout (Prn-KO) *B. pertussis* strains to characterize the specific effect of Prn production by this pathogen on DC maturation. DC cytokine production, surface marker expression, and transcriptional profiles were analyzed following bacterial stimulation. In addition to in vitro studies, we used a murine infection model to investigate the effect of Prn-deficient *B. pertussis* strains in vivo. Gene expression profiles in murine lungs and cytokine profiles in sera were determined at two time points post-infection. Together, these studies show that compared to the isogenic parental strains, Prn-KO *B. pertussis* strains induced increased production of pro-inflammatory cytokines by DCs and expression of genes involved in cell death upon murine infection. These findings indicate that *B. pertussis* strains not producing Prn can alter the immune response to this pathogen.

## Results

### Characterization of Prn-deficient *B. pertussis* mutant strains

**Fig. 1:**
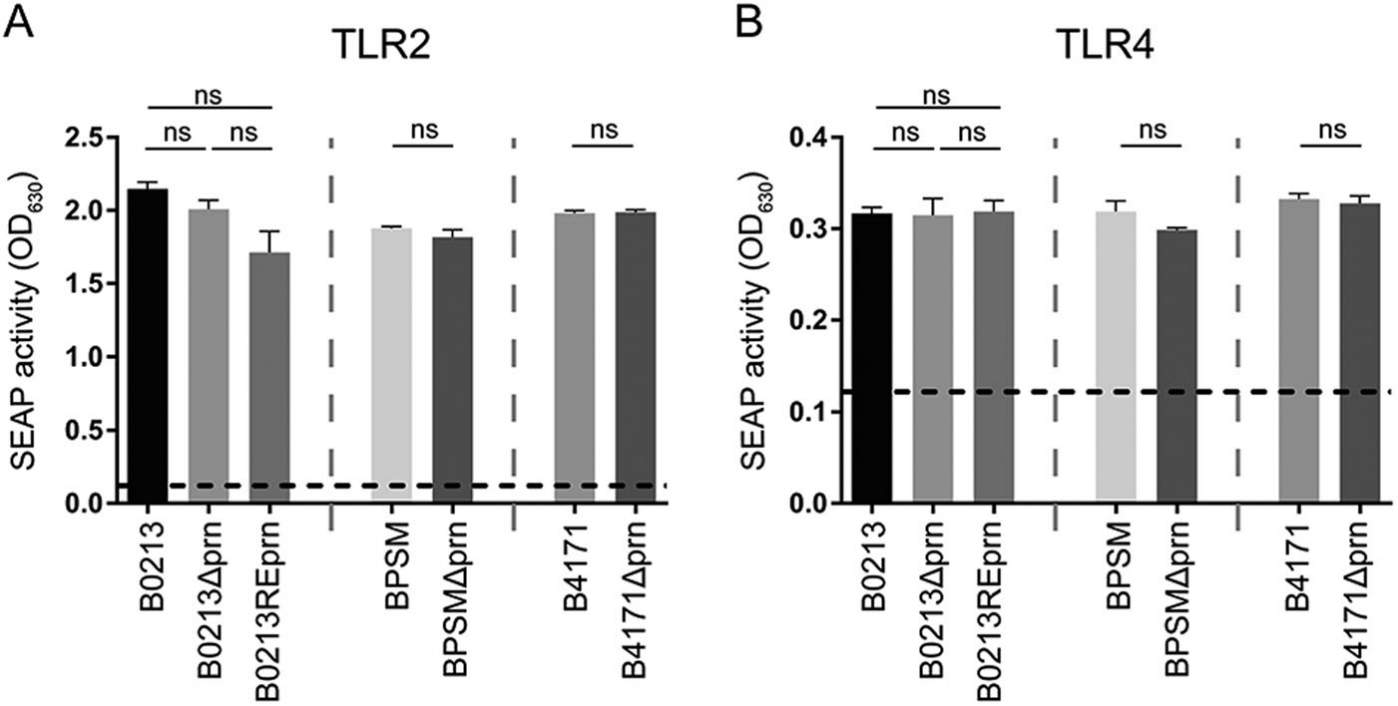
Characterization of strains used in this study. The different strains used in this study, B0213, B0213Δprn, B0213REprn, BPSM, BPSMΔprn, B4171, and B4171Δprn (MOI of 40), were used to stimulate HEK-Blue cells either expressing **a** TLR2 or **b** TLR4. Activation of these receptors is indicated as SEAP activity. Medium stimulation is indicated by the dotted line. Ns non-significant

A whole-cell enzyme-linked immunosorbent assay (ELISA) was used to determine the production of Prn and other virulence factors, namely FHA, Ptx, lipooligosaccharide (LOS), Fim2, Fim3, and Vag8, by B0213, B0213Δprn, B0213REprn, BPSM, BPSMΔprn, B4171, and B4171Δprn. As expected, Prn was absent in the three Prn-KO strains (B0213Δprn, BPSMΔprn, and B4171Δprn). The levels of all other virulence factors were comparable between parental and mutant strains (Table 1). The presence/absence of Fim2 or Fim3 corresponds to the strain genotype (Table 2). Additionally, PCR confirmed the *prn* deficiency (Figure S1). Moreover, mutant and parental strains induced similar levels of TLR2 and TLR4 activation in HEK-Blue reporter cells, suggesting comparable levels of LOS and lipoproteins/lipopeptides on parental and Prn-KO strains (Fig. 1a, b). Control experiments indicate no activation of the parental HEK-Null cell line upon stimulation with the different *B. pertussis* strains and the activation of HEK-TLR2 and HEK-TLR4 cells by the corresponding ligands (Figure S2).

**Table 1:** Whole-cell ELISA of *B. pertussis* virulence factors

	Virulence factors						
Strains	FHA	Prn	PTX	LOS	Fim2	Fim3	Vag8
B0213	3.6	0.4	0.5	1.8	1.5	0.1	1.0
B0213Δprn	3.8	0.0	0.5	1.8	1.5	0.1	0.8
B0213REprn	3.4	0.3	0.4	2.0	1.3	0.1	0.8
B4171	2.3	0.3	0.7	1.9	0.1	0.8	1.2
B4171Δprn	1.8	0.0	0.5	2.3	0.1	0.9	1.2
BPSM	1.6	0.3	0.4	2.1	0.7	0.1	1.1
BPSMΔprn	1.6	0.0	0.5	2.1	0.8	0.1	1.4

**Table 2:** Characteristics of strains used in this study

Strain	Genotype	Remarks	Reference
B0213	*ptxP1, ptxA2, prn1,Fim2*	Strep resistant Tohama I derivative	^14^
B0213Δprn	*ptxP1,ptxA2, prn::kan, Fim2*	Prn knock out derived from B0213	^13^
B0213REprn	*ptxP1, ptxA2, prn1, Fim2*	Restored expression mutant derived from B0213Δprn	^13^
BPSM	*ptxP1, ptxA2, prn1, Fim2*	Strep resistant Tohama I derivative	^15^
BPSMΔprn	*ptxP1,ptxA2,prn::deletion, Fim2*	Prn knock out derived from BPSM	This study
B4171	*ptxP3, ptxA1, prn2 Fim3*	Strep resistant 2014 clinical isolate	This study
B4171Δprn	*ptxP3, ptxA1, prn2::kan, Fim3*	Prn knock out derived from B4171	This study

Altogether, the *B. pertussis* Prn-KOs used in this study, besides the production of Prn, do not differ from the isogenic wild type strains in the production of well characterized bacterial virulence factors.

### *B*. *pertussis* Prn-KO B0213Δprn strain induces enhanced production of pro-inflammatory cytokines by moDC

**Fig. 2:**
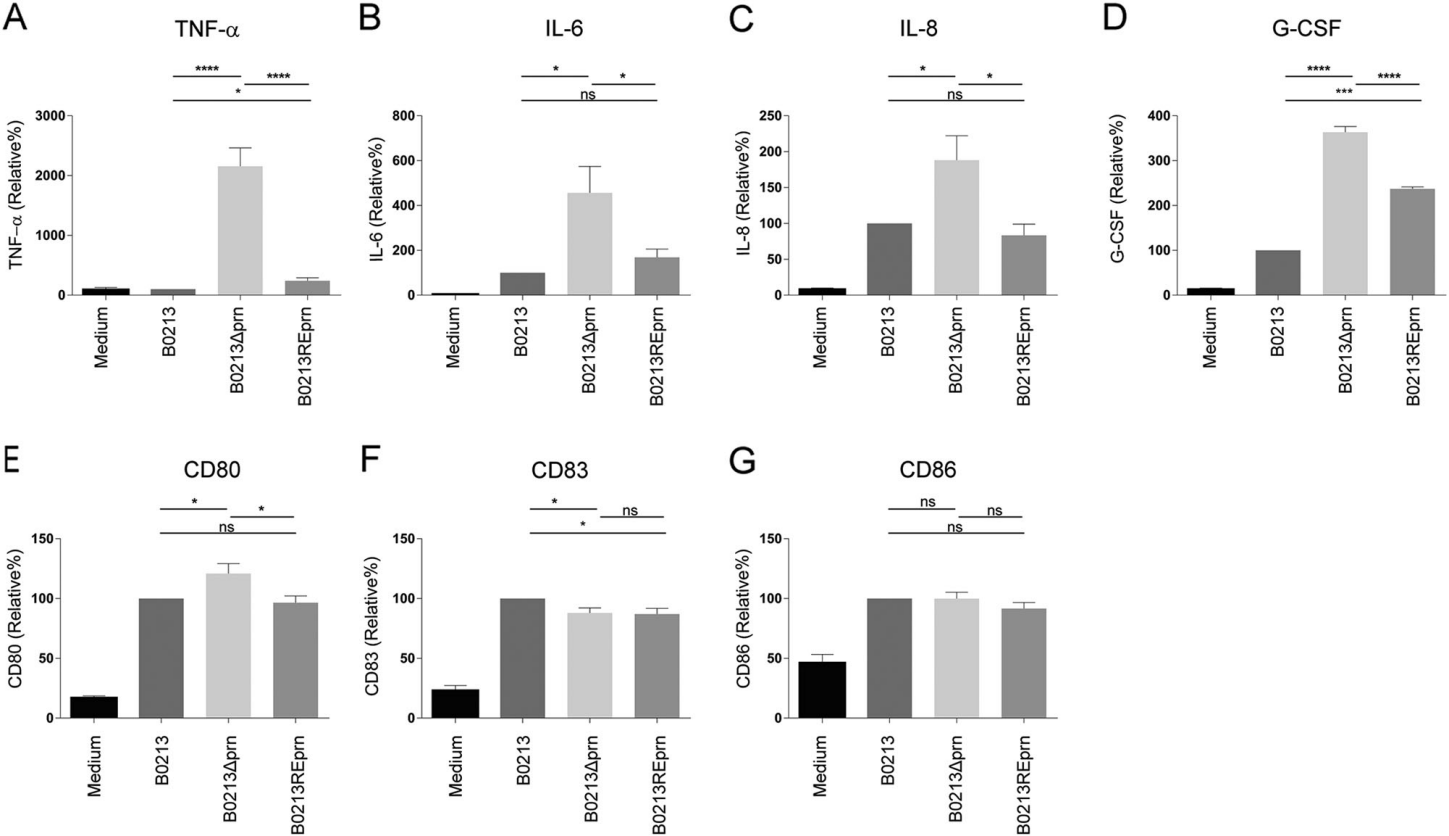
moDC stimulation with B0213Δprn induced increased pro-inflammatory cytokine production compared to B0213 or B0213REprn stimulation. MoDCs were stimulated with B0213, B0213Δprn or B0213REprn at an MOI of 10 or left unstimulated for 48 h. Using luminex, moDC production of **a** IL-6, **b** TNF-α, **c** IL-8, and **d** G-CSF in the supernatant and surface expression, using flow cytometry, of **e** CD80, **f** CD83, and **g** CD86 was determined. Experiments were performed using at least three donors *P ≤ 0.05, **P ≤ 0.01, ***P ≤ 0.001, ****P ≤ 0.0001

DCs are important innate immune cells which are instrumental for the induction of a protective adaptive immune response^13^. Human moDCs were used to investigate the effect of Prn production by *B. pertussis* on DC maturation by stimulating them for 48 h with the Tohama I strain B0213, B0213Δprn, and B0213REprn, in which the expression of the *prn* gene was restored. Compared to moDCs stimulated with the isogenic parental strain, results indicated that B0213Δprn induced a strong increase in TNF-α production, as well as a significant increase in IL-6, IL-8, and G-CSF production by moDCs (Fig. 2a–d). Upon stimulation with the restored mutant B0213REprn, the production of TNF-α, IL-6, IL-8, and G-CSF were either fully or partially restored to the levels induced by the parental strain. Using these three *B*. pertussis strains, we observed no significant differences in the levels of IL-12p70, IL-1β, Eotaxin, IP-10, MIP-1α, MIP-1β, MCP-1 produced by moDCs (data not shown). Moreover, flow cytometric analyses indicated only minor changes in the expression of surface maturation markers CD80, CD83, and CD86 on moDCs stimulated with the three different strains (Fig. 2e–g).

### *B. pertussis* B0213Δprn induces distinct gene expression profile in human DCs

**Fig. 3:**
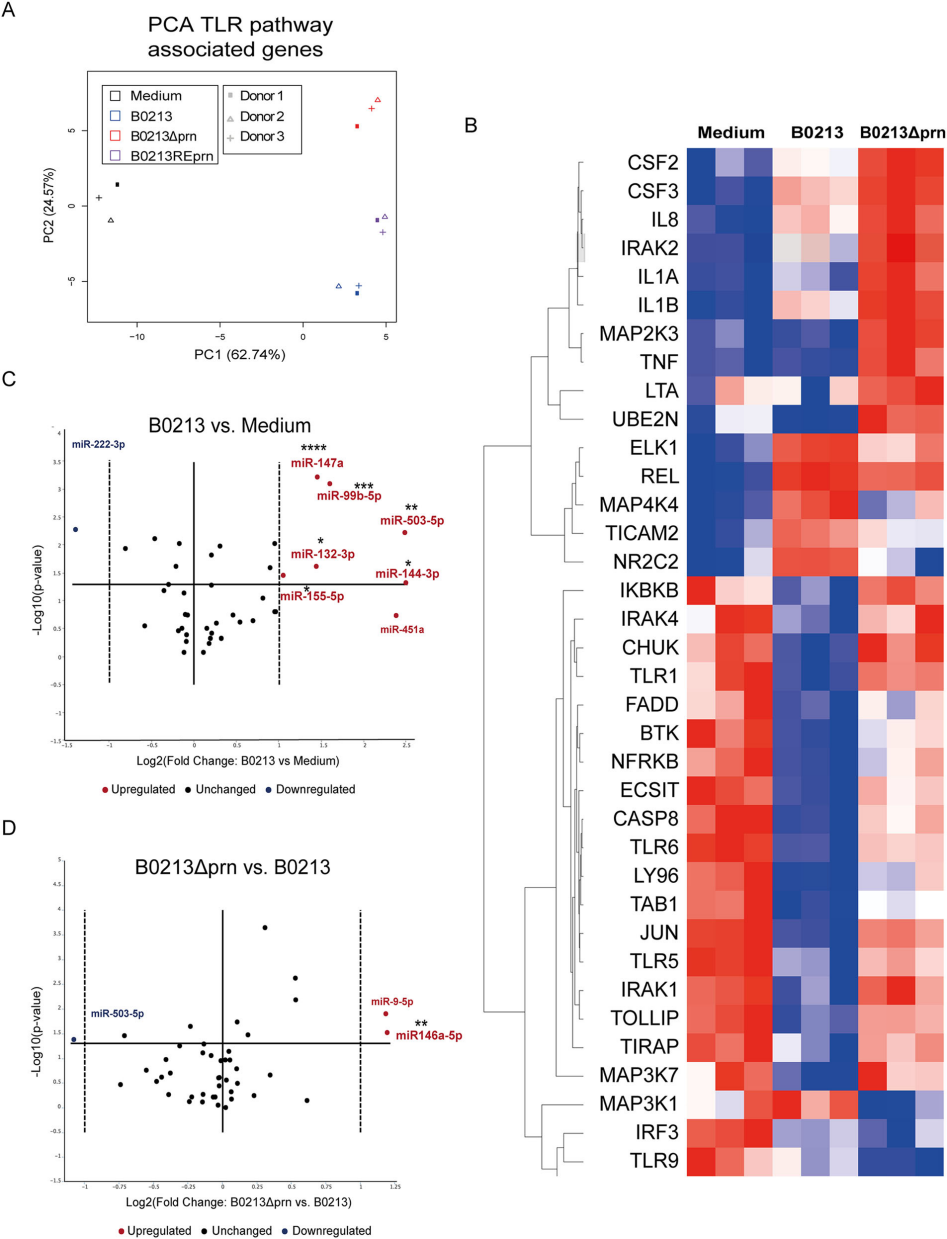
moDC stimulation with B0213Δprn induces a differential gene expression profile compared to B0213 or B0213REprn stimulation. RNA was isolated 6 h post infection and gene expression of 84 different TLR-pathway associated genes was analysed by means of a **a** PCA. Moreover, **b** the heatmap shows the hierarchical clustering (Pearson’s correlation) of relative expression levels for differentially expressed genes between medium, B0213, and B0213Δprn stimulated moDC. Relative expression levels for each individual gene are presented as minimum (blue) and maximum (red). Rows represent gene expression profiles of individual samples, 6 h after stimulation. For all samples and all genes included in the TLR-qPCR array. The expression of various microRNAs by moDCs was determined comparing **c** parental strain B0213- with medium-stimulated moDCs as well as **d** B0213Δprn- with B0213-stimulated moDCs. Experiments were performed using at least three donors. * *P* ≤ 0.05, **P ≤ 0.01, *** *P* ≤ 0.001, ****P ≤ 0.0001

To further characterize the phenotype of the moDCs induced upon stimulation with B0213, B0213Δprn or B0213REprn, gene expression levels 6 h post stimulation were analysed for 84 genes involved in the TLR-pathway using a qPCR array. A list of all the differently expressed genes can be found in Table S2, where 2-fold and higher upregulation and downregulation are marked in red and blue respectively. A principal component analysis (PCA) (Fig. 3a) on all 84 genes showed differences in gene expression profiles between medium and B0213 or B0213Δprn stimulated moDCs. Moreover, the gene expression induced by the B0213 and B0213REprn strains is comparable. This suggests that Prn production affects moDC gene expression. Genes involved in NF-kB signalling were significantly upregulated upon stimulation with the B0213 strain compared to medium stimulation. These include *IRAK2, MAPK4K, NFKB1/2, NFKBIA*, and *REL* as well as *PTGS2* and *TICAM1* (3.4, 7.2, 4.1, 3.5, 19.2, 232.9, and 2.5 fold respectively), which are known to be upregulated in response to bacterial stimulation^14,15^. The *CD80* gene expression levels were found to be increased compared to medium (32.1 fold) and similar among moDCs stimulated with the three strains, confirming the obtained flow cytometric data. Moreover, upon stimulation with B0213, moDCs show significantly decreased expression of *TLR1*, *TLR4*, *TLR5,* and *TLR6* compared to medium stimulated moDCs (−12.1, −4.7, −15.7, and −5 fold respectively).

We next focussed on the 36 genes that were significantly differently expressed between B0213 and B0213Δprn stimulated moDCs. The expression of these genes for all experimental conditions are depicted in a heat map (Fig. 3b) where, minimum (blue) and maximal (red) expression values are shown for each gene. Upon comparison of the gene expression following B0213 or B0213Δprn stimulation, results corroborate the findings on cytokine production (Figure S3); the gene expression levels of *TNF*, *IL8*, and *CSF3*, encoding for G-CSF, were significantly increased (39.9, 4 and 15.5 fold respectively) upon moDC stimulation with the B0213Δprn compared to B0213. Although not significant, the expression of the *IL6* also increased (5.7 fold) when moDCs were incubated with the B0213Δprn compared to B0213. Additionally, *IL1B* and *IL1A* expression (8.3 and 16.2 fold) was significantly increased upon stimulation with the B0213Δprn compared to B0213, as was the expression of *IRAK1/2*, *JUN,* and *MAP2K3* (2.1/3.2, 5, and 3.2 fold respectively) coding for downstream signalling molecules. Moreover, B0213Δprn induces significantly more expression of *TLR1*, *TLR5*, and *TLR6* (9.5, 4.7, and 2.4 fold respectively) and an increasing trend for *TLR2* (3.1 fold) compared to B0213. Together, these data indicate that the lack of Prn accounts for the increased expression of genes involved in the production of pro-inflammatory cytokines by moDCs.

We also determined the microRNAs (miRNA) expression of moDCs 24 h post stimulation for all three strains. For this purpose, we used a custom panel of 39 different microRNAs that we selected from literature based on their involvement in DC maturation and/or survival (Table S1). We found that compared to medium, moDCs stimulated with B0213 showed significantly increased expression of hsa-miR-132-3p, hsa-miR-144-3p, hsa-miR-147a, hsa-miR-155-5p, hsa-miR-503-3p, and hsa-miR-99b-5p and a decreased expression hsa-miR-222-3p (Fig. 3c). Notably, the expression of hsa-miR-146a-5p was significantly increased by moDCs stimulated with B0213Δprn (Fig. 3d) but not with B0213 or B0213REprn (data not shown).

Collectively, these data show a differential gene expression profile both on mRNA and miRNA level upon moDC stimulation with B0213Δprn compared to B0213 stimulation. In line with the observed findings on cytokine production, we observed a comparable gene expression profile between DCs stimulated with B0213 and the restored mutant B0213REprn.

### Immunomodulatory effect of Prn deficiency is independent of the *B. pertussis* strain background

**Fig. 4:**
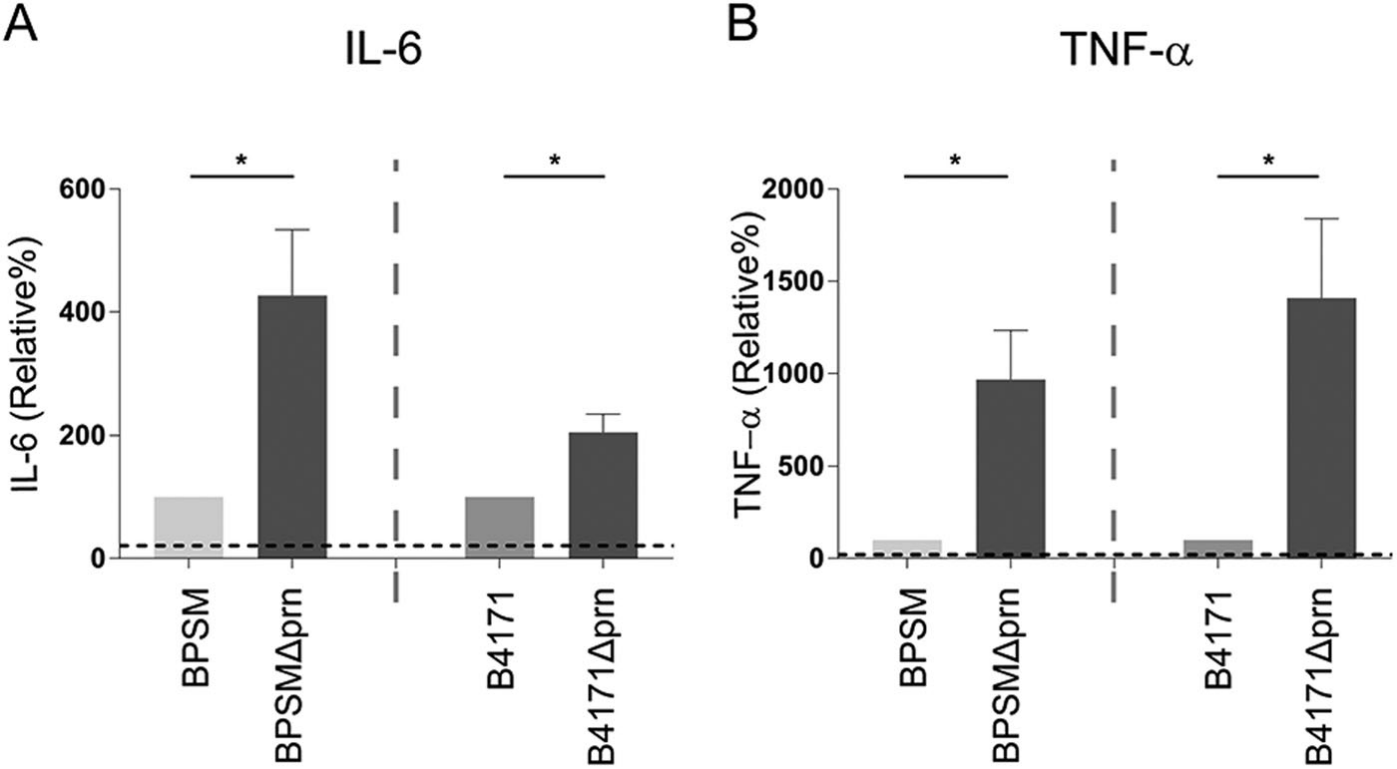
Prn deficiency in different bacterial backgrounds also induced an increased pro-inflammatory cytokine profile upon moDC stimulation. MoDCs were stimulated with BPSM, BPSMΔprn, B4171 or B4171Δprn at an MOI of 10 or left unstimulated for 48 h. MoDC production of **a** IL-6 and **b** TNF-α was determined in the supernatant. Experiments were performed using at least three donors *P ≤ 0.05

To determine whether a complete deletion of the *prn* gene renders comparable results as observed with B0213Δprn in which the *prn* gene is interrupted, we examined the Tohama I derivative BPSM and its Prn-deficient derivative BPSMΔprn, which has a complete deletion of the *prn* gene. We also examined the effect of another *B. pertussis* strain, namely B4171 which is a clinical strain carrying the pertussis toxin promotor allele (*ptxP*) 3 isolated in 2014 and its Prn-deficient derivative B4171Δprn. As shown in Fig. 4, moDCs stimulated with either BPSMΔprn or B4171Δprn also induced increased production of TNFα and IL-6 compared to moDC stimulated with the isogenic parental strains (Fig. 4a, b), further supporting the role of Prn in immune modulation.

### Infection with BPSMΔprn induces altered expression profiles in murine lungs

**Fig. 5:**
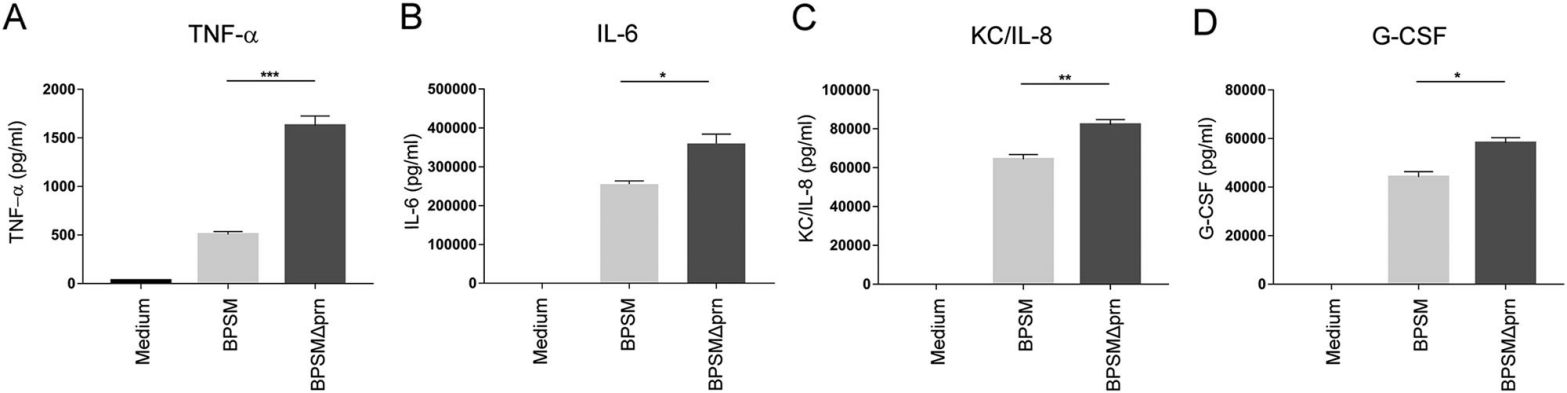
BPSMΔprn-stimulated murine BMDC show an increased production of pro-inflammatory cytokines. Murine BMDCs were stimulated with BPSM or BPSMΔprn at an MOI of 100 or left unstimulated for 48 h. BMDC production of **a** TNF-α, **b** IL-6, **c** KC/IL-8, and **d** G-CSF in the supernatant was determined. Experiments were performed using murine BMDCs from four mice per experimental condition *P ≤ 0.05, **P ≤ 0.01, ***P ≤ 0.001

**Fig. 6:**
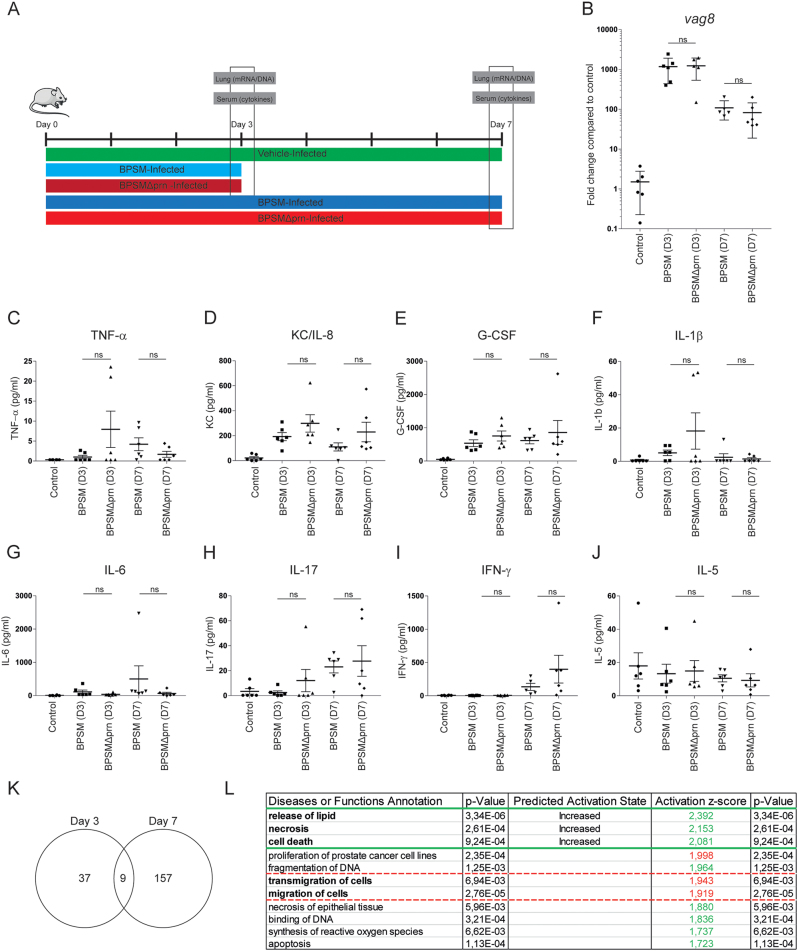
Murine infection with BPSMΔprn results in a different immune response compared to BPSM. **a** Balb/c mice were infected with 10^6^ CFU of the parental BPSM, BPSMΔprn or left uninfected as control. Serum for cytokine analysis and lungs for RNA sequencing were collected on day 3 and day 7 post infection. Bacterial DNA load in the lungs was determined using a **b** qPCR on the *Bordetella*-specific target gene *vag8* relative to the murine household gene *ptger2*. In serum of BPSM-, BPSMΔprn-infected and control mice, the presence of **c** TNF-α, **d** IL-6, **e** G-CSF, **f** IL-17, **g** KC/IL-8, **h** IL-1β, **i** IFN-γ, and **j** IL-5 was determined on both time points post-infection. **k** Venn diagram showing the amount of differentially expressed genes on day 3 and 7 in the lungs of mice infected with BPSMΔprn, as compared to BPSM infected mice, based on averaged normalized gene expression levels of groups. RNA sequence data on day 7 was analysed using the **l** diseases or function annotation tool of the software programme Ingenuity from Qiagen. The animal experiment was performed using six mice per infection group per time point and six control uninfected mice. *P ≤ 0.05, **P ≤ 0.01, ***P ≤ 0.001

To assess the in vivo relevance of Prn production by *B. pertussis*, we first determined whether the effect that Prn-KO strains have on human moDC could also be observed when using murine BMDCs. To this end, BMDCs were stimulated for 48 h with either BPSM or BPSMΔprn. As was observed for human moDCs, results indicated that compared to BPSM stimulation, BPSMΔprn induces a strong increase in TNF-α production, as well as a significant increase in IL-6, KC/IL-8, and G-CSF production (Fig. 5a–d). Next, mice were infected with BPSM or BPSMΔprn or left uninfected as control (Fig. 6a). Using qPCR with *vag8* as a *Bordetella*-specific target gene, we confirm the presence of BPSM or BPSMΔprn in the murine lungs on day 3 and 7 after infection (Fig. 6b). Sera and lungs were collected on days 3 and 7 post infection and used for cytokine determination and gene expression profiling, respectively (Fig. 6a). Although not reaching statistical significance, average serum levels of TNF-α, KC/IL-8, G-CSF and IL-1β on day 3 post-infection where higher in BPSMΔprn- compared to BPSM-infected mice (Fig. 6c–f). No differences in IL-6, IL-17, IFN-y, and IL-5 were observed (Fig. 6g–j). On day 7, we observed a similar trend as on day 3 for KC/IL-8 (Fig. 6d) and for IFN-y (Fig. 6i). For all the other cytokines no differences were observed.

The lung gene expression of control mice and those infected with either BPSM or BPSMΔprn was additionally monitored on day 3 and day 7 using RNAseq. Compared to control mice, lungs of mice infected with BPSM had 6860 genes differentially expressed on day 3 and 7831 genes on day 7. A list of all differentially expressed genes on both time points can be found in Table S3. As gene expression profiles following *B. pertussis* infection have already been extensively described by others^16^, we focused on the genes that were differentially expressed in BPSM versus BPSMΔprn infected mice. On day 3, differential expression between the groups was identified for 46 genes. On day 7, 166 differentially expressed genes were found (Table S4, Fig. 6k). Of these genes, nine were detected on both days (Fig. 6k). To determine the biological processes involved, the differentially expressed genes were analyzed using Ingenuity Pathway Analysis. By making use of the ‘Diseases and Functions’ tool we show compared to BPSM, a significant increase in genes (indicated by a z-score of above 2) annotating to the release of lipids, necrosis, and cell death after infection with BPSMΔprn on day 7 (Fig. 6l). Additionally, we detected a decrease in genes annotated to the migration of cells, almost reaching the z-score cut off of 2 (Fig. 6l).

Altogether, findings indicate that murine infection with BPSMΔprn compared to BPSM shows an increasing trend in pro-inflammatory cytokine detection in serum. Moreover, on day 7, genes related to the release of lipids, necrosis and cell death are increasingly expressed in the lungs of mice infected with BPSMΔprn compared to BPSM, indicating that the absence of Prn in *B. pertussis* alters the lung gene expression profile of these animals.

## Materials and methods

### Ethics statement

This study was conducted according to the principles described in the Declaration of Helsinki and for the collection of samples and subsequent analyses, all blood donors provided written informed consent. Blood samples were processed anonymously and the research goal, primary cell isolation, required no review by an accredited Medical Research Ethics Committee (MREC), as determined by the Dutch Central Committee on Research involving human subjects (CCMO). Regarding animal procedures, all experiments were carried out in accordance with the guidelines of the French Ministry of Research, and the protocols were approved by the Ethical Committee of the Region Nord-Pas-de-Calais in France.

### Bacterial strains and growth conditions

Selected *B. pertussis* strains were inoculated onto Bordet Gengou (BG) agar plates, supplemented with glycerol and 15% defibrinated sheep blood (BD Biosciences, Franklin Lakes, New Jersey, USA), and grown at 35 °C and 5% CO_2_ for 4 days. The strains used in this study are listed in Table 2. All mutants, with the exception of BPSMΔprn, were constructed as previously described^17^. Briefly, the plasmid pSR2-1.1 was used to introduce the 1 kB kanamycin resistance (*kan)* gene by allelic exchange into *prn1* of B0213^18^ (B0213Δprn also known as B1686) and B4171 (B4171Δprn). Also by allelic exchange, pSR2.1 was used to restore expression of *prn* (B0213REprn, also known as B2576) in the B0213Δprn background. The BPSMΔprn strain was constructed using the suicide vector pSS4940 (a pSS4245 derivative kindly provided by professor Stibitz) carrying flanking regions upstream and downstream of the *prn* gene, which resulted in a clean deletion of the entire *prn* gene (2733 bp) in BPSMB^19^. Successful deletion of *prn* and insertion of the *kan* gene were confirmed by PCR (Figure S1). Bacteria used in these experiments were suspended using a cotton swab in phosphate-buffered saline (PBS) (Gibco, New York, NY, USA) to collect bacteria from the BG agar plates. Bacterial concentration were determined by measuring the optical density (OD) at 600 nm.

### Determination of virulence factors produced by the B. pertussis strains using whole-cell ELISA

To measure the production of various virulence factors by the different *B. pertussis* strains, bacterial suspensions were prepared as described above, and heat inactivated at 56 °C for one hour. A 96 wells Nunc plate (Thermo Fischer Scientific, Waltham, MA, USA) was coated with 0.1 OD_600_ of the indicated bacterial preparations at room temperature. Control coatings were *B. pertussis* antigens: filamentous hemagglutinin (FHA), Prn, pertussis toxin (Ptx), LOS and fimbria3 sero-type 2 and 3 (Fim2/3) or outer membrane vesicles (OMVs). After overnight incubation at 37 °C, the ELISA was performed using monoclonal antibodies directed against Prn^20^, Ptx, FHA, Vag8, LOS or Fim2/3 derived from a hybridoma bank^21^. HRP-conjugated goat anti-mouse IgG (Southern Biotech, Brimingham, AL, USA) was added and the reaction was visualized by the addition of chromogenic TMB substrate (Thermo Fischer Scientific). The reaction was stopped by the addition of H_2_SO_4_ and absorbance was measured at 450 nm. The presence of *B. pertussis* antigens from the different strains are indicated as a ratio that was obtained by dividing the OD of the strains by the OD of the respective control antigen. The concentration of the control antigens used was 0.25 µg/mL for FHA, Prn, Ptx, LOS, and Fim2/3, giving an OD value of 0.94, 3.04, 1.07, 0.65, 1.42, and 2.84. For Vag8, 1.2 µg/mL OMV was used as control antigen giving an OD value of 1.16.

### Cell lines, culture, and stimulation

Human NF-κB/SEAP reporter HEK293 cells (HEK-Blue) transfected either with human TLR2 (HEK-Blue-hTLR2) or human TLR4 (HEK-Blue-hTRL4) in combination with MD-2 and CD14, as well as untransfected (HEK-Blue-Null1) cells (InvivoGen, San Diego, CA, USA), were grown in complete Dulbecco’s modified Eagle’s medium (DMEM, Gibco) supplemented with 10% heat-inactivated fetal calf serum (FCS, Thermo Scientific), 50 U/mL penicillin (Gibco) and 50 μg/mL streptomycin (Gibco) (HEK-Blue culture medium) as previously described with selection antibiotics^12^. Live bacteria (multiplicity of infection (MOI) of 40) were used to stimulate 2.5 × 10^4^ HEK-Blue-hTLR2, HEK-Blue-hTLR4, or HEK-Blue-Null1 cells for 22 h. Supernatant was collected and secreted embryonic alkaline phosphatase (SEAP) activity was determined using the QUANTI-Blue assay.

### Purification, culture, and stimulation of human moDCs

Monocytes were purified from peripheral blood derived from healthy donors as described previously^22^. Briefly, peripheral blood mononuclear cells were isolated by Lymphoprep (Nycomed, Zurich, Switzerland) gradient centrifugation at 1000 × *g* for 30 min. Subsequently, monocytes were collected by using MACS in combination with anti-CD14 microbeads (Miltenyi Biotech, Bergisch Gladbach, Germany). The isolated cells were 95–99% CD14+ as determined by flow cytometry. Monocytes were cultured in 24-well culture plates at 400,000 cells/well for 6 days at 37 °C in a humidified atmosphere containing 5% CO_2_ in Iscove’s Modified Dulbecco’s Medium (IMDM) supplemented with 1% FCS, 100 units penicillin, 100 units streptomycin, and 2.92 mg/mL L-glutamine (Gibco; DC culture medium), 500 U/mL human GM-CSF (PreproTech, Rocky Hill, NJ, USA) and 800 U/mL human IL-4 (Active Bioscience, Hamburg, Germany). On day 6, the immature moDCs were stimulated with the indicated live *B. pertussis* (MOI of 10) or left unstimulated in DC culture medium supplemented with 500 U/mL GM-CSF or 48 h for cytokine determination or 6/24 h for RNA isolation. Supernatants were filtered over a 0.22 μm filter to remove bacteria before cytokine determination (Millipore, Darmstadt, Germany).

### Purification, culture, and stimulation of murine BMDCs

BMDCs were isolated as described previously^23^ with minor modifications. In brief, bone marrow was flushed from the femur and tibia of Balb/c mice (Envigo, Horst, The Netherlands). Subsequently, cells were passed through a 70 µm nylon cell strainer, washed and resuspended in RPMI 1640 containing 10% FCS, 2mM–L-glutamine,100 U/mL streptomycin, 100 mg/mL penicillin (Gibco) and 50 μM β-mercaptoethanol. Cells were seeded in 6 wells plate at a density of 1 × 10^6^ cells per well in the presence of 20 ng/mL of murine GM-CSF (Biolegend, San Diego, CA, USA) and incubated at 37 °C, 5% CO_2_ for 7 days with fresh mGM-CSF added on day 2 and day 5. On day 7, cells were stimulated with live *B. pertussis* bacteria (MOI of 100) or remained unstimulated for 48 h.

### Flow cytometry analysis

Purity of the CD14-positive cells was determined by staining the monocytes with anti-CD14-PE (BD Biosciences). After 48 h of stimulation, moDCs cells were washed and resuspended in FACS-buffer (PBS pH 7.2; 0.5% BSA; 0.5 mM EDTA). Subsequently, the cells were stained with a panel of fluorochrome-conjugated monoclonal antibodies specified for moDC maturation (CD80, CD83, CD86 (BD Biosciences)) and with LIVE/DEAD Fixable Aqua Dead Cell Stain Kit (Invitrogen, Waltham, MA, USA) for 30 min at 4 °C. Following washing of the cells and fixation using 1.5% paraformaldehyde (PFA), cells were acquired on the FACS Canto II (BD Biosciences) and analyzed using FlowJo software (Tree Star, Ashland, OR, USA).

### Animal experiment

Six-week old female Balb/c mice were intranasally infected with 10^6^ viable BPSM or BPSMΔprn as described previously^24^ or received PBS as control (*n* = 6 per group). Mice were euthanized either at 3 or 7 days post-infection and blood was collected prior to harvesting the lungs. Total blood was left to coagulate and centrifuged at 1000 ×* g* for 5 min to collect sera, which was stored at −80 °C until further analysis. The left lung lobe was preserved in RNAlater (Thermo Scientific) and frozen at −80 °C for subsequent RNA isolation.

### Cytokine measurement by ELISA and luminex

The concentration of various cytokines and chemokines (IL-1β, IL-6, IL-8, IL-10, IL-12 (p70), G-CSF, TNF-α, Eotaxin, IP-10, MCP-1 (MCAF), MIP-1α and MIP-1β) in supernatants of moDC was determined using Bio-plex Pro cytokine kits (Bio-Rad, Hercules, CA, USA) according to the manufacturer’s instructions. The presence of TNF-α, G-CSF, IFN-γ, IL-1β, IL-4, IL-5, IL-6, IL-17, and KC (IL-8) in supernatants collected from murine BMDCs and sera was determined using a mouse milliplex MAP kit (Millipore). Measurements and data analysis were performed with the Bio-Plex 200, using Bio-Plex Manager software (version 6.1, Bio-Rad Laboratories).

### RNA isolation, qPCR, and sequencing

Total RNA was extracted from the moDCs, cultured in the presence of the indicated stimuli 6 h or 24 h post stimulation, for messenger RNA (mRNA) or microRNA (miRNA) analysis respectively. The Phenol extraction column-based miRNeasy RNA isolation kit (Qiagen, Venlo, the Netherlands) was used. Murine lung tissue collected on day 3 and day 7 after infection was homogenized by using the FastPrep®-24 bead homogenizer (MP Biomedicals, Santa Ana, CA, USA) in 700 µL Qiazol (Qiagen). RNA was isolated according to the manufacturer’s instructions with minor modifications, namely an extra column-washing step was added using 80% ethanol before extracting the RNA using RNase-free water. RNA quality and integrity was determined using Lab-on-Chip analysis on an Agilent 2100 Bioanalyzer (Agilent Technologies, Santa Clara, CA, USA). The RNA integrity numbers (RIN) of all RNA samples were >8.

To measure gene (mRNA) expression of moDCs, cDNA was synthesized from 200 ng of RNA using the First strand RT2 kit (Qiagen). The obtained cDNA was used in combination with the RT2 SYBR Green PCR kit (Qiagen) for the Toll-Like Receptor signaling pathway RT2 profiler PCR array (#PAHS-018Z, Qiagen) which contains 84 genes involved in TLR-mediated signal transduction and innate immunity, as well as all necessary controls, using a StepOnePlus™ Real-Time PCR System (Applied Biosystems, Foster City, CA, USA). For miRNA determination, cDNA was synthesized from 200 ng of RNA by means of the miScript II RT kit (Qiagen). The obtained cDNA was used in combination with the miScript SYBR Green PCR kit (Qiagen) and the two-step plus qPCR (Applied Biosystems) to run custom panels of miRNAs (#CMIHS02331C, Qiagen) containing 39 miRNAs selected from the literature for miRNAs known to be involved in DC maturation or survival and all necessary controls. An overview of the selected miRNAs can be found in Table S1. Data were analyzed by an online PCR data analysis platform provided by Qiagen (http://pcrdataanalysis.saviosciences.com/rna).

In order to confirm the presence of *B. pertussis* in the lungs of mice, the gene encoding for the *Bordetella* specific virulence associated gene 8 *vag8* was quantified from genomic DNA extracted from the lungs of infected and control mice employing a 7500 Fast thermal cycler using SYBR Green PCR Master Mix (Applied Biosystems). The reason why we chose *vag8* to detect the presence of *B. pertussis* in the lungs of the infected mice is because the gene for this virulence factor is present in *Bordetella spp*. Recently, we have unraveled the underlying mechanisms of complement evasion by Vag8 of *B. pertussis*^25^. Thermal cycling parameters consisted of 1 min at 50 °C and 10 min at 95 °C, followed by 40 cycles of 15 s at 95 °C and 1 min at 60 °C using the following primers: Vag8-sense (GGT TCA CTG GTA GAG AGC AC), Vag8-anti-sense (GTT GAG CAG GGA CAC ATT AC), murine GAPDH-sense (TGC ACC ACC AAC TGC TTA G) and murine GAPDH-anti-sense (GGA TGC AGG GAT GAT GTT C). Gene levels were quantified according to the following formula: 2^-(Cti-Cta)^ where C_ti_ the threshold cycle of the gene of interest and C_ta_ is the threshold cycle of murine GAPDH.

Gene expression profiles in the murine lungs were measured by RNAseq. Libraries for the Illumina platform were generated using the TruSeq Stranded mRNA Library Prep Kit using 1 µg of total RNA as input. Briefly, the mRNA fraction was purified from total RNA by polyA capture, fragmented and subjected to first-strand cDNA synthesis with random hexamers in the presence of Actinomycin D. Second-strand synthesis was performed incorporating dUTP instead of dTTP. Barcoded DNA adapters were ligated to both ends of the double-stranded cDNA and subjected to PCR amplification. Libraries were subsequently checked on a Bioanalyzer (Agilent) and quantified using qPCR with Kapa-kit (Roche, Basel, Switzerland). The libraries were pooled at equimolar concentrations and sequenced on an Illumina HiSeq 3000 (single end, 50 bp reads). The sequencing run was analyzed with the Illumina CASAVA pipeline (v1.8), and demultiplexed FASTQ files were generated based on sample-specific barcodes (>15 million reads/sample). RNAseq reads were aligned to the mouse reference genome (UCSC mm10) and the most recent transcript annotations using STAR (v.2.5.0b). Expression levels of all transcripts were quantified and normalized using Cufflinks (2.1.0) and expressed as Fragments Per Kilobase of transcript per million mapped reads (FPKM). Differentially expressed genes (DEG) were determined by Cuffdiff and considered significantly different with false discovery rate (FDR) < 0.1. The data have been deposited in the National Center for Biotechnical Information Gene Expression Omnibus (GEO)^26^ and are accessible through GEO series with the preliminary accession code GSE35610. GEO Processes were identified using Ingenuity Pathway Analysis (Build version: 441680M).

### Statistical analysis

Statistical significance was determined by using ANOVA followed by unpaired *t*-tests for the moDC and BMDC experiments. FDR was controlled at the level of 10% by applying the Benjamini–Hochberg method to the results of all the tests performed. Results that passed a selection based on the FDR of 0.1. *P*-values ≤ 0.05 were considered statistically significant.

## Discussion

Whooping cough, caused by the Gram-negative respiratory pathogen *B. pertussis*, is a re-emerging disease. This re-emergence has been attributed to a variety of factors, including pathogen adaptation that occur most likely due to vaccination pressure^27^. For example, the vaccine strains carry the *ptxP2* and *ptxP1* alleles as opposed to the *ptxP3* allele which is currently carried by over 90% of the circulating strains worldwide^27^. Another striking and most recent feature is the loss of production of antigens that are present in the ACV. This is especially evident for the autotransporter protein Prn. In ACV-using countries, such as Australia and the United States of America, over 80% of *B. pertussis* strains isolated from patients no longer produce Prn^6,28^. In The Netherlands, where the ACV was introduced around 10 years later than in the United States of America, 15% of the recently isolated strains were reported to be Prn-deficient^12^. Due to the association with ACV-usage, Prn deficiency may be the result of the inclusion of the highly immunogenic Prn antigen in the vaccine^29^. *B. pertussis* strains not expressing one or more of the other vaccine antigens have also been reported^30,31^. Nonetheless, these isolates are less commonly circulating than the Prn-deficient strains. This raises questions regarding the function of Prn in *B. pertussis* infection and the effect of Prn-deficient *B. pertussis* strains on the immune response.

Studies characterizing immune responses to Prn-deficient *B. pertussis* strains are limited and have not used isogenic strains, a major drawback, making it difficult to uncover the specific effects of Prn. Most studies are dedicated to evaluating the fitness of Prn-producing and Prn-deficient *B. pertussis* strains during infection of vaccinated mice. This has indicated enhanced colonization of Prn-deficient strains in ACV-immunized mice, suggesting that the rise of Prn-deficient strains is most probably due to vaccine pressure^29,32^. In addition to our recent observations^12^, only one more study compared the effect of one Prn-deficient and a Prn-expressing strain on moDC maturation^33^. The authors found no difference in the DC maturation profile upon stimulation with these *B. pertussis* strains and an increased invasiveness of moDCs by the Prn-deficient clinical isolate compared to the non-related reference strain ATCC 97-97^33^. The latter is a reference strain known to have an altered LOS structure which has been associated with decreased TLR4 activation^22^. Therefore, the exclusive effect of Prn production by *B. pertussis* on moDC activation remains unclear. DCs will, depending on the mode of activation, produce different cytokines and other immune mediators which will promote the differentiation of specific T helper (Th) responses necessary for protection against pathogens. For example, the production of IL-12p70 and IL-23 is essential for differentiation of Th1 and cytotoxic T cells or Th17 cells respectively and these subsets have been reported to be important for a protective immune response against *B. pertussis*^34,35^. Therefore, it is important to study the effect that different *B. pertussis* strains have on DC maturation. To our knowledge, the present study is the first to directly assess the effect that the absence or presence of Prn production by *B. pertussis* has on the DC phenotype using multiple Prn-deficient strains and their respective isogenic strains.

Our findings indicate that stimulation of moDCs with *B. pertussis* strains deficient in Prn results in an increased production of pro-inflammatory cytokines by these cells. This DC phenotype was observed when stimulating these cells with three different Prn-KO strains, either in a Tohama I as well as in the recent (2014) *B. pertussis* clinical isolate B4171 background. These findings suggest that Prn has an immunomodulatory function, dampening the production of pro-inflammatory cytokines. Prn produced by the closely related *Bordetella bronchiseptica* has previously been suggested to have an immunomodulatory role during infection^36^. Prn of *B. pertussis* and *B. bronchiseptica* have a sequence identity of over 90% suggesting that a comparable protein function is highly probable^37^.

Expression of 84 genes involved in the TLR signaling pathway was assessed in moDCs stimulated with B0213, B0213Δprn or B0213REprn. As expected, the expression of genes encoding pro-inflammatory cytokines was upregulated in B0213Δprn-stimulated moDCs corroborating our cytokine production data. Moreover, a difference was found in the expression of TLRs which were downregulated upon stimulation with B0213 compared to the medium control. This is not surprising as TLR expression in innate cells has been shown to decrease over time upon microbial stimulation^38^. This could result in diminished responsiveness of DCs to microorganisms, a strategy described for bacteria of the gut microbiota^39^. Low levels of TLR2, which can form a heterodimer either with TLR1 or TLR6, and low levels of TLR5 have been associated with reduced responsiveness of Langerhans cells, the DCs of the skin, to bacteria^40^. Of notice is that the *TLR1*, *TLR5*, *TLR6*, and *TLR2* genes were expressed at higher levels in B0213Δprn compared to B0213 stimulated moDCs. Future studies including the characterization of downstream signalling upon stimulation of DC with these strains may clarify the observed differences.

In addition to the expression of genes involved in the TLR pathway, we found that compared to B0213, B0213Δprn-stimulated moDCs increased their expression of the miRNA mir-146a-5p. Expression of this miRNA has been shown to be upregulated by DCs following stimulation with *Helicobacter pylori*^41^ and has, in line with our findings, been associated with increased pro-inflammatory cytokine secretion^42^. This miRNA has also been reported to induce apoptosis in DCs^43^. Interestingly, our in vivo experiments do not only show a minor increase of pro-inflammatory cytokines in serum of BPSMΔprn– vs BPSM-infected mice but more importantly, the analysis of the RNA sequence data of the murine lungs revealed a significant increase in genes belonging to functional groups related to cell death. Whether apoptosis and/or necrosis contribute to the induced cell death remains to be investigated. As it is difficult to pinpoint which cells are responsible for these observed effects, one must be cautious to interpret data from a complete organ. We speculate that the increased induction of pro-inflammatory cytokines by DCs, such as high levels of TNF-α, which can mediate cell death^44^, and miRNA146a-5p, could be in large part responsible for the increased expression of genes involved in cell death in the murine lungs.

Many pathogens, including *B. pertussis*, have been shown to modulate DC responses to enhance bacterial survival^45,46^. Different strategies have been described, including our recent findings indicating that unlike older strains, recently circulating Prn producing strains induced a dominant IL-10 production by moDC^12^. Here, we made use of *B. pertussis* Prn knock-out and respective isogenic strains, which allowed us to investigate the exclusive effect of the absence of Prn production by *B. pertussis* on the immune response. The data generated suggests an immunomodulatory role for Prn. Unlike the Prn-KO strains used in this study, Prn-deficient, compared to Prn-producing, clinical isolates do not appear to differ in their effect on DC maturation^12^. It is likely that during the evolutionary process towards Prn deficiency, other mutations in the bacterial genome compensate for the loss of Prn. This work sets the basis for further research directed towards elucidating the mechanism of action of Prn and identifying these potential compensating mechanisms of the currently circulating Prn-deficient *B. pertussis* strains.

## Electronic supplementary material

Supplemental Figure 1(TIF 1160 kb)

Supplemental Figure 2(TIF 342 kb)

Supplemental Figure 3(TIF 297 kb)

Supplemental table 1(XLSX 12 kb)

Supplemental table 2(XLSX 27 kb)

Supplemental table 3(XLSX 756 kb)

Supplemental table 4(XLSX 21 kb)

Supplemental Materials(DOCX 14 kb)
