# Supplementary figures and images for: Bordetella pertussis pertactin knock-out strains reveal immunomodulatory properties of this virulence factor
Source: Emerg Microbes Infect. 2018 Mar 21;7:39. doi: 10.1038/s41426-018-0039-8 (PMC5861065; doi:10.1038/s41426-018-0039-8)

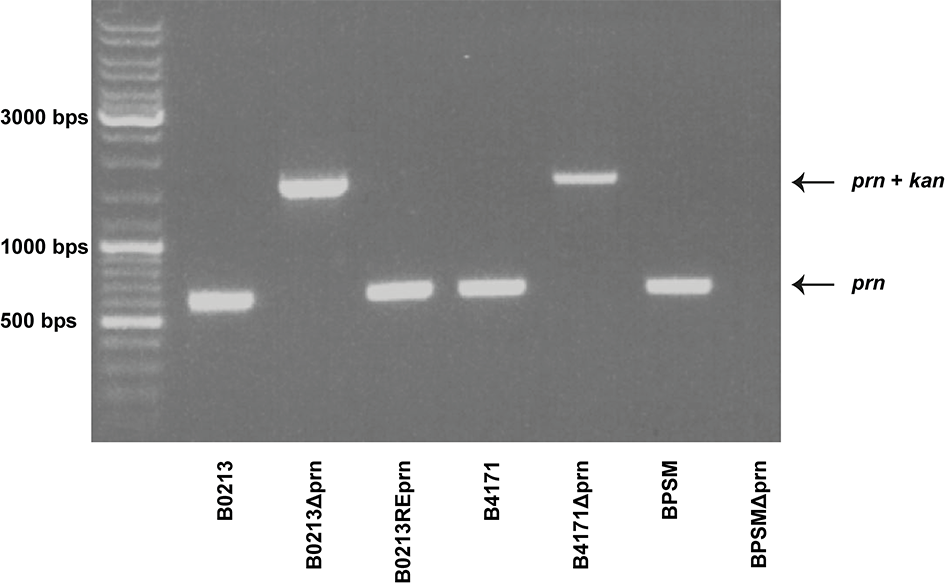

Supplement: Supplementary file 1 — Supplemental Figure 1(TIF 1160 kb) [file 41426_2018_39_MOESM1_ESM.tif]

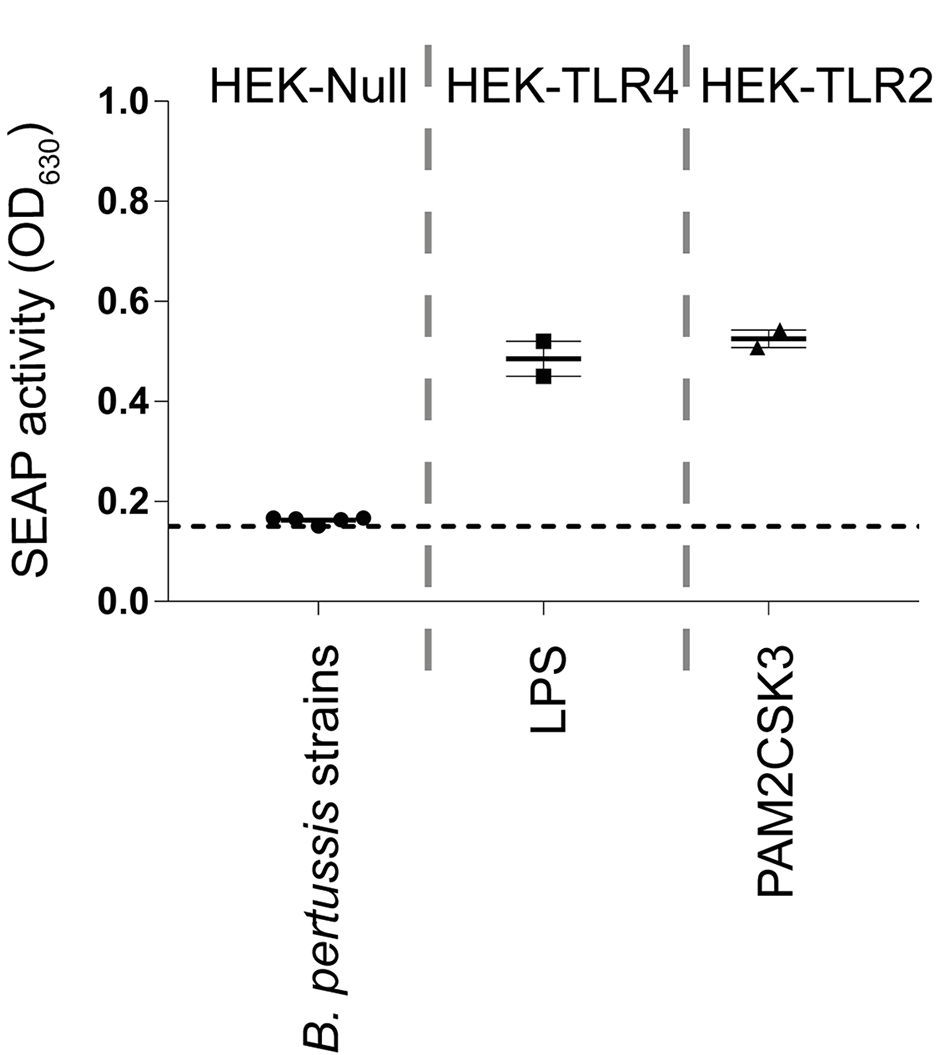

Supplement: Supplementary file 2 — Supplemental Figure 2(TIF 342 kb) [file 41426_2018_39_MOESM2_ESM.tif]

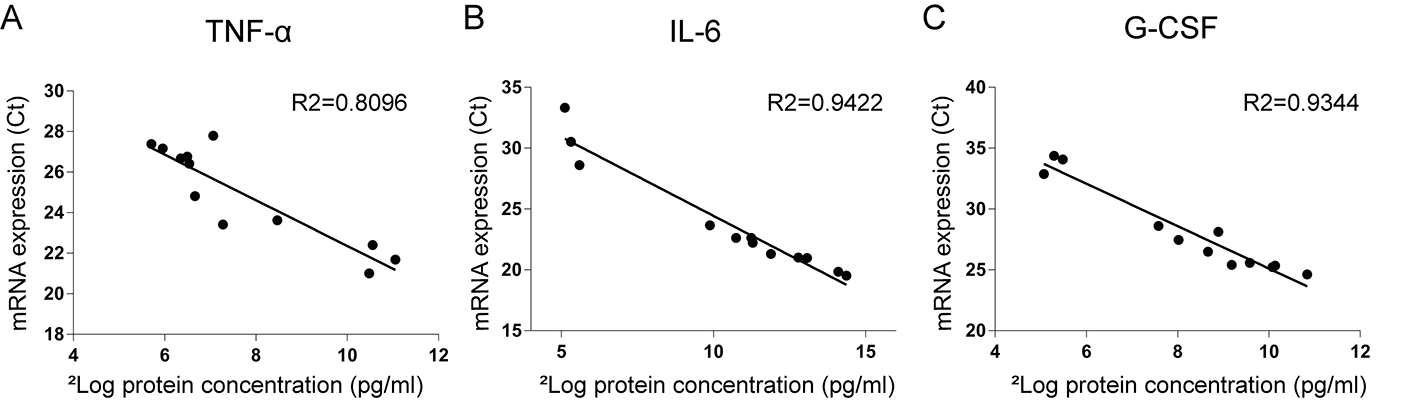

Supplement: Supplementary file 3 — Supplemental Figure 3(TIF 297 kb) [file 41426_2018_39_MOESM3_ESM.tif]
