# Supplementary material for: Bordetella pertussis pertactin knock-out strains reveal immunomodulatory properties of this virulence factor
Source: Emerg Microbes Infect. 2018 Mar 21;7:39. doi: 10.1038/s41426-018-0039-8 (PMC5861065; doi:10.1038/s41426-018-0039-8)
Supplement: Supplementary file 8 — Supplemental Materials(DOCX 14 kb) [file 41426_2018_39_MOESM8_ESM.docx]

**Figure S1 Determination of the presence or absence of the *prn* gene in all strains used in this study** Using PCR, the insertion of the *kan* cassette to interrupt the *prn* gene or total deletion of the *prn* gene was determined. The Agarose gel shows the Prn-PCR product and confirms the presence of *prn* in B213, BPSM and B4171, *kan* insertion or lack of *prn* for B0213Δprn, B4171Δprn and BPSMΔprn respectively and restoration of *prn* for B0213REprn.

**Figure S2** **Controls for the HEK cell experiment.** The parental HEK-Null cells were stimulated with the strains used in this study to control for aspecific activation (left panel represented as dots). Activation of HEK-TRL4 (middle panel, squares) and HEK-TLR2 (right panel, triangles) cells with the respective ligands LPS and PAM2CSK3. The dotted lines indicate medium values.

**Figure S3** **Correlation between mRNA expression and protein concentration.** The correlation between mRNA expression at 6 hours post stimulation and protein concentration 48 hours of TNF-α, IL-6 and IL-8 after stimulation was assessed by linear regression. R^2^ values are indicated in the graphs.

**Supplementary tables:**

Supplemental Table 1 Selected microRNAs for the qPCR array.

Supplemental Table 2 Differentially expressed genes of moDC using the TLR-pathway associated genes qPCR array comparing B213, B0213Δprn and B0213REprn.

Supplemental Table 3 Differentially expressed genes in murine lungs comparing BPSM infected mice to uninfected control mice infected mice at 3 and 7 days post infection.

Supplemental Table 4 Differentially expressed genes in murine lungs comparing BPSM infected mice to BPSMΔprn infected mice at 3 and 7 days post infection.
